# Platelet-rich plasma and hyaluronic acid in the treatment of acute ankle sprains: A review

**DOI:** 10.17305/bb.2025.13327

**Published:** 2025-12-03

**Authors:** Yu-Tung Chen, Wen-Tien Wu, Ru-Ping Lee, Tzai-Chiu Yu, Ing-Ho Chen, Kuang-Ting Yeh

**Affiliations:** 1 School of Medicine, Tzu Chi University, Hualien, Taiwan; 2 Department of Orthopedics, Hualien Tzu Chi Hospital, Buddhist Tzu Chi Medical Foundation, Hualien, Taiwan; 3 Institute of Medical Sciences, Tzu Chi University, Hualien, Taiwan; 4 Graduate Institute of Clinical Pharmacy, Tzu Chi University, Hualien, Taiwan

**Keywords:** Ankle injuries, platelet-rich plasma, hyaluronic acid, regenerative medicine, sprains and strains

## Abstract

Ankle sprains are prevalent musculoskeletal injuries commonly encountered in the general population, particularly among athletes. While conventional treatments are widely practiced, regenerative therapies have emerged as potential adjunctive options. This narrative review aims to assess the role of regenerative therapy in the management of acute ankle sprains and evaluate its efficacy through an analysis of the literature. We focused on studies available in PubMed, restricting our search to English-language articles published between January 2005 and December 2024. Our review identified five studies on platelet-rich plasma (PRP) and one on hyaluronic acid (HA). The PRP studies included four clinical trials and one case report. PRP injections demonstrated short-term benefits in pain reduction and functional recovery, particularly when administered early and in multiple doses. However, long-term outcomes were often comparable to standard treatments or placebo. The study on HA indicated consistent and sustained advantages over placebo in alleviating pain, expediting the return to sport, and reducing recurrence rates. Based on the current evidence, PRP and HA may function as adjunctive therapies for acute ankle sprains, especially for short-term symptom relief and functional recovery. Treatment efficacy appears to be influenced by factors such as injection timing, volume, immobilization protocols, and the concurrent use of nonsteroidal anti-inflammatory drugs. Nonetheless, the evidence base remains constrained by small sample sizes, heterogeneous protocols, and a lack of long-term follow-up. Therefore, further high-quality randomized controlled trials are essential to establish standardized protocols and ascertain the long-term efficacy of these regenerative therapies.

## Introduction

Ankle sprains are prevalent musculoskeletal injuries, particularly among individuals participating in sports, with nearly 40% of all traumatic ankle injuries occurring in this context [1]. The most common mechanism of injury involves a combination of foot inversion and adduction during plantar flexion, which can compromise the lateral ankle ligaments, including the anterior talofibular ligament (ATFL), calcaneofibular ligament (CFL), and posterior talofibular ligament (PTFL) [2]. The ATFL is recognized as the weakest and most frequently injured ligament within the lateral ankle complex. Biomechanical studies indicate that the ATFL possesses the lowest tensile strength and ultimate load to failure among all lateral ligaments [3]. Furthermore, approximately 40% of individuals who experience an acute ankle sprain subsequently develop chronic ankle instability (CAI), which can result in recurrent sprains, avoidance of sports activities, or even early onset of osteoarthritis in the ankle [4, 5]. This highlights the necessity for timely management of acute ankle sprains to prevent long-term complications. However, this review will not address the treatment of established CAI, focusing instead on regenerative therapies administered during the acute phase of ankle sprains, typically within the initial weeks post-injury. Mentions of CAI within this manuscript are limited to contextual background on its epidemiology and natural history, without informing treatment recommendations or conclusions.

Effective management of acute ankle sprains is crucial in preventing recurrent injuries and the development of CAI [6]. Ankle sprains are classified into three grades based on severity: Grade I involves mild ligament stretching without rupture or instability; Grade II includes partial ligament tears, characterized by moderate pain, swelling, and some joint instability; and Grade III denotes complete ligament rupture, marked by severe pain, swelling, bruising, functional loss, and significant joint instability [7].

Evidence-based clinical guidelines outline various treatment modalities for acute ankle sprains, including the Rest, Ice, Compression, Elevation (RICE) protocol, non-steroidal anti-inflammatory drugs (NSAIDs), functional treatment, and surgical interventions [1]. While NSAIDs are commonly prescribed to manage pain in patients with acute ankle sprains, their use may hinder the natural healing process, as the inflammation they suppress plays a critical role in tissue recovery [8]. Functional support, such as ankle braces or tape, is frequently utilized in managing acute ankle sprains, with recommendations for 4–6 weeks of support over immobilization. Among the options for functional support, ankle braces have demonstrated the highest effectiveness [9]. Surgical intervention may be necessary to reduce the incidence of recurrent lateral ankle sprains (LASs), as repeated sprains can elevate the risk of developing osteoarthritis. Although surgery yields favorable clinical outcomes for both chronic injuries and acute complete lateral ligament ruptures, functional treatment is generally preferred, as not all patients require surgical intervention [10]. The management strategy for ankle sprains is contingent upon injury severity. Grade I sprains are typically addressed conservatively with the RICE protocol, early mobilization, and functional rehabilitation, including strengthening and proprioceptive exercises. Grade II sprains necessitate a longer period of protected weight bearing, often utilizing a walking boot or brace, along with controlled range-of-motion exercises and structured physical therapy to restore function and prevent chronic instability. In cases of Grade III sprains, surgical intervention may be warranted for persistent instability, particularly in high-demand athletes. Post-immobilization rehabilitation focuses on regaining strength, proprioception, and joint stability. Across all grades, pain management with NSAIDs is common, and treatment plans should be individualized based on the patient’s activity level and clinical progress [7].

Despite the availability of evidence-based guidelines offering various treatment options for acute ankle sprains, the role and efficacy of regenerative therapies remain insufficiently defined within clinical practice. Regenerative approaches, specifically platelet-rich plasma (PRP) and hyaluronic acid (HA) injections, have garnered increased clinical interest due to their biological potential to enhance tissue healing. PRP contains concentrated growth factors that may facilitate ligament repair and modulate inflammation, while HA is proposed to promote tissue healing through its anti-inflammatory properties [10]. Nevertheless, despite their growing clinical application, the evidence base for these interventions in acute ankle sprains remains fragmented and inadequately synthesized. To date, a systematic review has specifically evaluated PRP for acute ankle sprains [11], highlighting potential benefits while noting substantial variability in preparation protocols, injection timing, and outcome measures; the study concluded that high-quality evidence and standardized protocols are essential. However, this review focused solely on PRP and did not consider other regenerative modalities, such as HA. Importantly, no focused narrative synthesis exists that specifically examines regenerative injection therapies administered during the acute phase (typically within the first few weeks) of injury, identifies key factors influencing treatment efficacy, or offers practical clinical guidance for implementing these therapies in practice. Therefore, this narrative review aims to address these knowledge gaps by: (1) systematically reviewing the available clinical evidence for regenerative injection therapies—specifically PRP and HA—during the acute phase of ankle sprains; (2) identifying factors that may influence treatment efficacy, including injection timing, volume, and concurrent interventions; (3) synthesizing current evidence regarding short-term and long-term clinical outcomes; and (4) providing practical clinical insights to inform decision-making for patients with acute ankle sprains.

## Materials and methods

A literature search was conducted in PubMed on December 15, 2024, encompassing publications from January 2005 to December 2024. Limiting the search to a single database may have introduced selection bias and potentially overlooked relevant studies indexed in other databases, such as Embase, Web of Science, or the Cochrane Library. Nevertheless, PubMed was chosen due to its status as the most comprehensive database for biomedical literature and its free accessibility, which enhances the reproducibility of our search strategy for other researchers. The search strategies employed were: (1) (platelet-rich plasma [Title/Abstract] OR PRP [Title/Abstract]) AND (ankle sprain [Title/Abstract] OR lateral ankle sprain [Title/Abstract] OR ankle ligament injury [Title/Abstract]); and (2) (hyaluronic acid [Title/Abstract] OR HA [Title/Abstract] OR sodium hyaluronate [Title/Abstract]) AND (ankle sprain [Title/Abstract] OR lateral ankle sprain [Title/Abstract] OR ankle ligament injury [Title/Abstract]). We restricted our search to Title/Abstract fields rather than full-text to maintain specificity and focus on studies where regenerative therapies for ankle sprains were the primary focus. While this approach may have reduced sensitivity, it ensured that retrieved articles were directly relevant to our research question. No additional filters were applied to the database search. Two independent reviewers (Y-TC and K-TY) screened all titles and abstracts for eligibility in duplicate, working independently and blinded to each other’s decisions. Articles deemed potentially relevant by either reviewer underwent full-text assessment, which was also performed independently and in duplicate. Any disagreements between the reviewers during the title/abstract screening or full-text assessment stages were resolved through discussion, and if necessary, consultation with a third reviewer (C-YC) to reach consensus.

The inclusion criteria for literature selection were as follows: (1) studies involving patients diagnosed with acute ankle sprain (defined as injury occurring within the past six weeks); (2) clinical studies evaluating the efficacy of regenerative therapies, such as PRP or HA, in treating acute ankle sprains; (3) original clinical research articles, including randomized controlled trials, prospective or retrospective studies, and case reports with detailed clinical documentation; (4) studies reporting relevant clinical outcomes, such as pain relief, functional recovery, or other measures of treatment effectiveness; (5) articles published in English; and (6) studies published between January 2005 and December 2024. The exclusion criteria included: (1) studies focusing on CAI or other chronic ankle conditions rather than acute ankle sprains; (2) non-clinical studies, including animal models, *in vitro* experiments, or basic science research; (3) review articles, systematic reviews, meta-analyses, and expert opinions; (4) studies that did not involve regenerative therapy or did not specifically evaluate treatment effects for acute ankle sprains; (5) articles published in languages other than English; and (6) studies without full-text availability or incomplete data.

Despite advancements in translation tools, we restricted study inclusion to English-language publications to ensure consistency and reliability in data extraction and interpretation, minimizing potential misinterpretation of specialized clinical terminology, treatment protocols, or outcome measures. We acknowledge that this language restriction may have excluded potentially relevant studies published in other languages, particularly from regions where regenerative therapies are actively investigated, representing a limitation of this review.

A total of 15 articles were initially identified through the database search, comprising nine articles related to PRP and six related to HA. After removing duplicates and screening titles and abstracts, 10 articles underwent full-text assessment, of which four articles were excluded (two focused on CAI rather than acute ankle sprains, one was a review article, and one was a basic science study without clinical outcomes). Ultimately, six studies were included in the narrative review: five articles on PRP [12–16] (four clinical trials and one case report) and one on HA [17] (a randomized controlled trial) (Table 1). This systematic approach to literature selection, although limited by a single-database search and language restriction, ensured that all included studies were relevant to the research question and met the predefined quality standards for inclusion in this narrative review.

**Table 1:** Summary of studies evaluating regenerative therapies for acute ankle sprains

**Literature**	**Method**	**Indication for injection**	**Sample size**	**Injected material**	**Times of injection**	**Duration between injection and injury**	**Injected volume** **(mL)**	**Concentration**	**Immobilization time after injection**	**Outcome evaluation methods**	**Results**
Zhang et al. [12]	Clinical trial	First time Grade II LAS	83	PRP (intra-ligament)	2	48 h/4 weeks	3–4	6 folds	2 weeks	AOFAS and VAS	Early symptom relief
Blanco-Rivera et al. [13]	RCT	First-time Grade II LAS	21	PRP (intra-ligament)	1	Not reported	5	Not reported	10 days	AOFAS and VAS	Early symptoms and functional relief
Laver et al. [14]	RCT	Elite athletes with AITFL tears	16	PRP (intra-ligament)	2	Initial/7 days	1.5	2–3 folds	Not reported	RTP	Shorter RTP time and less residual pain
Lai and Sit [15]	Case report	LAS with ATFL complete tear	1	PRP (intra-ligament)	1	11 days	3	Not reported	4 weeks	Dynamic ultrasound images and MRI	Sonography and MRI confirmed healing
Rowden et al. [16]	RCT	Severe ankle sprains	37 enrolled, 33 analyzed	PRP (intra-ligament)	1	Not reported	5–6	Not reported	3 days	VAS and LEFS	No significant difference
Petrella et al. [17]	Clinical trial	Competitive athletes with Grade I/II ankle sprains	158	HA (periarticular)	2	Within 48 h/ day 4	0.7–1.2	MW 750–1000 kDa, 20 mg	Dosing per physician protocol	VAS	Reduced pain, faster recovery

Note: ATFL and AITFL are anatomically distinct ligaments. The ATFL is a component of the lateral ligament complex, frequently injured in inversion ankle sprains. In contrast, the AITFL is part of the syndesmotic ligament complex, which stabilizes the distal tibiofibular joint. Abbreviations: PRP: Platelet-rich plasma; AOFAS: American Orthopedic Foot and Ankle Society; VAS: Visual analog scale; RCT: Randomized controlled trial; AITFL: Anterior inferior tibiofibular ligament; ATFL: Anterior talofibular ligament; RTP: Return-to-play; LAS: Lateral ankle sprains; MRI: Magnetic resonance imaging; NSAIDs: Non-steroidal anti-inflammatory drugs; LEFS: Lower Extremity Functional Scale; HA: Hyaluronic acid; MW: Molecular weight; kDa: Kilodalton.

### PRP application for the treatment of acute ankle injury

This review examines five articles related to PRP therapy for the management of acute ankle injuries, comprising four clinical trials and one case report. Among the clinical trials, three were randomized, while one was not.

Zhang et al. [12] conducted a clinical trial to evaluate the impact of PRP injections on clinical outcomes and the healing quality of ATFL in patients with grade II LASs. Eighty-three patients experiencing their first LAS were divided into three groups: a no-injection group, a group receiving a single PRP injection within 48 h of injury, and a group receiving two PRP injections (one at 48 h and another at 4 weeks). PRP was administered under ultrasound guidance, and all ankles were immobilized for two weeks. Clinical outcomes were assessed using the American Orthopedic Foot and Ankle Society (AOFAS) and visual analog scale (VAS) scores at 2, 6, 8, 24, and 48 weeks. The quality of the ATFL was evaluated using magnetic resonance imaging (MRI)-based signal-to-noise ratio (SNR) at 8, 24, and 48 weeks. Results indicated that the PRP injection groups experienced greater pain relief and improved functional outcomes compared to the control group, with the two-injection group demonstrating the most significant improvements at 8 weeks. However, at the 6- and 12-month follow-ups, clinical outcomes were similar across all groups. MRI findings revealed enhanced healing quality of the ATFL over time, with the two-injection group exhibiting the best SNR results at the final follow-up. In conclusion, PRP injections provided early symptom relief for patients with LASs, with two injections leading to superior short-term clinical outcomes and ATFL healing. However, long-term recovery was comparable across groups.

Blanco-Rivera et al. [13] performed a randomized clinical trial to assess the clinical effects of PRP therapy in patients with acute grade II LASs treated with rigid immobilization. Twenty-one first-time sprain patients were included, all receiving rigid immobilization for 10 days. The experimental group received a PRP injection over the ATFL prior to immobilization. Pain and functional outcomes were evaluated using the VAS, AOFAS scores, and the Foot and Ankle Disability Index at 3, 5, 8, and 24 weeks. The PRP group exhibited greater pain reduction and superior functional scores at 8 weeks compared to the control group. However, at 24 weeks, both groups demonstrated similar clinical outcomes.

Laver et al. [14] conducted a randomized trial to investigate the effects of ultrasound-guided PRP injections on the recovery and dynamic stability of elite athletes with syndesmotic (high ankle) sprains involving tears of the anteroinferior tibiofibular ligament. Sixteen athletes were randomized into a PRP treatment group (*n* ═ 8) or a control group (*n* ═ 8), both following identical rehabilitation and return-to-play (RTP) protocols. Clinical outcomes, pain levels, and dynamic ultrasound assessments were conducted at baseline and 6 weeks post-injury. The PRP group had a significantly shorter RTP time (40.8 ± 8.9 days) than the control group (59.6 ± 12.0 days, *P* ═ 0.006). Additionally, PRP-treated athletes reported significantly less residual pain, with only one patient (12.5%) experiencing minor discomfort upon resuming activity, compared to five patients (62.5%) in the control group. One patient in the control group required syndesmotic reconstruction due to persistent pain and disability.

Lai and Sit [15] presented a case study of a 39-year-old runner who sustained a high-grade LAS with a complete tear of the ATFL. Following initial treatment with oral analgesics and rest, the patient pursued PRP therapy for expedited recovery. A single PRP injection was administered under ultrasound guidance, followed by four weeks of immobilization in a cast. Ultrasonography at 4 weeks revealed no ligament gapping, and the cast was subsequently removed. At 8 weeks, the patient resumed jogging, with ultrasonography confirming ligament healing. By 6 months, the patient was pain-free and ran daily, with MRI confirming complete ATFL healing. This case supports the efficacy of PRP as a promising nonsurgical option for recovery from high-grade LASs.

Rowden et al. [16] conducted a prospective, randomized, double-blind, placebo-controlled trial to evaluate PRP therapy for severe ankle sprains in an emergency department setting. Out of 1156 screened patients, 37 met the inclusion criteria and were enrolled, with four patients withdrawing before the injection procedure. The remaining 33 patients were randomized to receive either a PRP injection (*n* ═ 18) or a placebo (normal saline, *n* ═ 15). All participants completed the study protocol with no loss to follow-up. Both groups received a single injection followed by three days of immobilization in a posterior splint. Outcomes were assessed using VAS and Lower Extremity Functional Scale (LEFS) at days 0, 3, 8, and 30. No statistically significant differences were observed between groups in pain scores or functional outcomes at any time point, indicating that PRP did not confer additional benefits over placebo for acute ankle sprains.

### HA application for the treatment of acute ankle injuries

Only one randomized clinical trial met our criteria regarding HA treatment. Petrella et al. [17] conducted a randomized controlled prospective trial involving 158 competitive athletes with grade I or II acute LASs. Participants were randomly assigned within 48 h of injury to receive either a periarticular HA injection (molecular weight 750–1000 kDa, 20 mg, volume 0.7–1.2 mL, administered using anatomical landmarks) plus standard care (the RICE protocol) or a placebo injection plus standard care. The HA injections were administered within 48 h of injury and were repeated on day 4. Follow-ups were conducted on days 30, 90, and 712. Assessments at baseline and on days 4, 8, 30, 90, and 712 included evaluations of pain during weight-bearing and a 20-meter walk (measured using a VAS), patient-reported severity of the ankle injury, satisfaction with treatment, time to pain-free and disability-free return to sports, recurrence of ankle sprains, missed sports days, and adverse events (AEs). The HA group exhibited significantly lower VAS scores for pain at all follow-ups compared to the placebo group (*P* < 0.001). The HA group achieved pain-free and disability-free return to sports earlier (11 ± 8 days) than the placebo group (17 ± 8 days, *P* < 0.05). At 24 months, the HA group experienced fewer recurrent ankle sprains (7 vs 16, *P* < 0.05), fewer missed sports days (21 vs 41, *P* < 0.002), and higher patient satisfaction at all time points. No serious AEs were reported. Thus, compared with placebo treatment, periarticular HA injections administered using anatomical landmarks were shown to be highly effective and well-tolerated, resulting in reduced pain, faster recovery, fewer recurrent ankle sprains, and fewer missed days from sports, with sustained benefits for up to 24 months.

## Discussion

This review exclusively examined regenerative therapies for acute ankle sprains, defined as injuries occurring within six weeks. Although CAI has been identified as a potential long-term complication of inadequately treated acute sprains, and knee osteoarthritis (KOA) studies are discussed in an analogous context, evidence regarding CAI and KOA was not utilized to inform our treatment recommendations. These recommendations are solely based on the six ankle-sprain-specific studies identified in this review. Consequently, the following discussion and conclusions pertain exclusively to the acute phase of ankle sprain management. Additionally, we acknowledge that our 20-year search window (2005–2024) was specifically selected to capture the seminal study by Petrella et al. [17] on HA, which remains the only high-quality randomized controlled trial evaluating HA for acute ankle sprains and is frequently cited in contemporary literature. Furthermore, we included one case report [15] due to its comprehensive ultrasound and MRI documentation of ATFL healing following PRP treatment. Given the extremely limited evidence base for regenerative therapies in acute ankle sprains, we believe these inclusions provide valuable clinical insights while maintaining methodological transparency and rigor. The case report was clearly labeled in our data presentation, and its limitations in evidence level have been appropriately discussed in the context of the overall findings.

Our recent narrative review evaluated the literature on regenerative interventions for acute ankle sprains available on PubMed. Among the six articles reviewed, five pertained to PRP and suggested that it may help patients achieve improved short-term clinical outcomes, as assessed using AOFAS, VAS, and RTP scores [12–15]. However, a randomized clinical trial conducted by Rowden et al. found that PRP did not confer any additional benefits over placebo. Several studies reported no significant differences in age or sex between the experimental and control groups [12–16]. A study by Laver et al. [14] differed slightly from the others; it focused on athletes with high ankle sprains (syndesmotic sprains), including soccer, rugby, and basketball players, judokas, and downhill mountain bikers. The results indicated not only a shorter RTP time but also reduced discomfort compared to the control group. Petrella et al. [17] also targeted athletes in their study, investigating the effects of HA treatment, where primary outcomes showed benefits in terms of VAS score reduction. Compared to the general population, athletes often have access to more structured and intensive rehabilitation programs for acute ankle sprains, potentially contributing to the positive outcomes observed in athlete-focused studies [18]. Furthermore, PRP and HA therapies may serve as adjunct treatments for acute ankle sprains, as most studies included additional interventions, such as the RICE protocol and cast immobilization [12].

We analyzed various factors, including the number of injections, injection sites, injection volume, total blood volume collected, PRP concentration, time elapsed after the sprain prior to injection, and duration of immobilization, to assess their impact on treatment outcomes (Table 1). The number of injections varied among studies, with some administering a single injection and others opting for two. The timing of these injections post-sprain also differed, especially in studies with two injections. For instance, Zhang et al. [12] administered the second injection four weeks after the first, while Laver et al. [14] administered it just one week later. Despite these variations, both studies reported symptomatic relief. The volume of whole blood collected ranged from 20 to 50 mL, with injected volumes between 0.7 and 6 mL. In Rowden et al. [16], NSAIDs were excluded during treatment, whereas Petrella et al. [17] utilized NSAIDs as part of standard care, including 500 mg of acetaminophen as a rescue medication. Regarding PRP concentration, only Zhang et al. [12] and Laver et al. [14] reported specific increases, with concentrations rising six-fold and two- to three-fold, respectively. Most studies administered injections directly into the ligament under ultrasound guidance; however, Blanco-Rivera et al. [13] and Rowden et al. [16] employed alternative methods, injecting PRP under the lateral malleolus and at the site of maximum tenderness, respectively. The duration of immobilization varied, ranging from 3 days to 4 weeks. Notably, in Rowden et al. [16], which reported no significant differences in outcomes, the immobilization period was the shortest at just 3 days, significantly less than in other trials [19]. This suggests that shorter immobilization periods may influence the efficacy of PRP and HA injections.

PRP preparations have gained prominence across various medical fields due to their potential to enhance tissue repair [20]. The underlying principle of PRP therapy is that concentrated platelets, when injected at injury sites, release biologically active factors, including growth factors, cytokines, lysosomes, and adhesion proteins [21]. These factors initiate the hemostatic cascade, stimulate the synthesis of new connective tissue, and promote revascularization [22]. The primary advantages of PRP include its safety, autologous origin (derived from the patient’s blood), and versatile preparation techniques, allowing for diverse medical applications [20]. Compared to corticosteroids, PRP presents a lower risk of adverse effects, although post-injection pain, swelling, and rare infections remain possible [23]. Nevertheless, the lack of clear regulations regarding the formulation and composition of PRP injections results in significant variability in platelet content, white blood cell counts, red blood cell contamination, and growth factor concentrations [24]. Additionally, medications such as NSAIDs can impact the release of the platelet secretome [25]. NSAIDs, commonly used to alleviate pain and inflammation in musculoskeletal disorders [26], function by inhibiting cyclooxygenase (COX) enzymes, thereby modulating the arachidonic acid pathway [27]. Aspirin irreversibly acetylates COX enzymes, leading to permanent inhibition throughout the platelet’s lifespan, while most non-aspirin NSAIDs (e.g., ibuprofen and naproxen) act as reversible COX inhibitors, producing transient effects on platelet function [27]. This inhibition disrupts placental growth factor signaling [28] and suppresses the production of key cytokines, including platelet-derived growth factor, fibroblast growth factor, vascular endothelial growth factor, and interleukins (IL-1β, IL-6, and IL-8), while elevating tumor necrosis factor-α levels [29]. Due to these effects on platelet-derived mediators, NSAIDs were generally avoided or not recommended in the reviewed PRP studies [12–16] due to concerns that NSAID-mediated suppression of inflammation and platelet function might hinder the regenerative healing cascade. Conversely, NSAIDs were permitted in Petrella et al.’s HA trial [17], where 500 mg of acetaminophen was available as rescue analgesia, reflecting the distinct non-platelet-dependent mechanism of HA. However, limited data exist regarding the molecular effects of NSAIDs on PRP efficacy in clinical contexts. Further research is needed to determine whether avoiding NSAIDs genuinely enhances PRP outcomes or if NSAIDs can be safely used alongside PRP as they can with HA [25].

**Figure 1.**
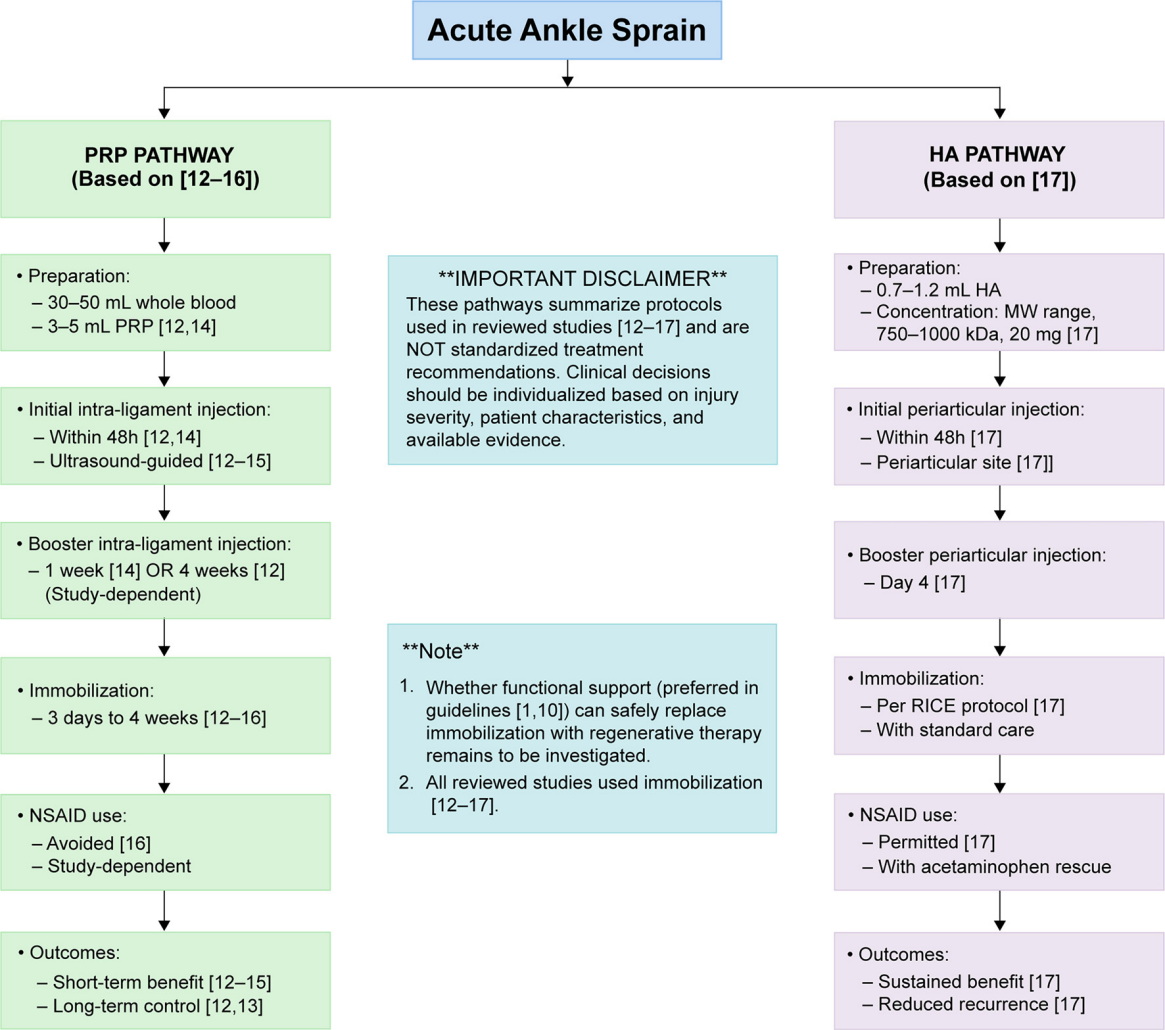
**Comparison of PRP and HA injection protocols for acute ankle sprains.** This figure summarizes the treatment protocols employed in the reviewed studies for PRP [12–16] and HA [17] injections in cases of acute lateral ankle sprains. The flowchart details preparation methods, injection timing, booster schedules, duration of immobilization, policies regarding NSAIDs, and reported outcomes. It is important to note that these pathways reflect the protocols from the studies and do not constitute standardized treatment recommendations. Clinical decisions should be tailored to individual patient needs. Additionally, while all reviewed studies included immobilization, the safety and efficacy of functional support—recommended in current guidelines [1, 10]—in conjunction with regenerative therapy warrant further investigation. Abbreviations: PRP: Platelet-rich plasma; HA: Hyaluronic acid; NSAID: Non-steroidal anti-inflammatory drug; RICE: Rest, ice, compression, elevation.

Based on the findings, we developed a hypothesis-generating treatment algorithm for acute ankle sprains (Figure 1). This algorithm is not intended as a clinical practice guideline or standardized treatment recommendation but serves as a synthesis of the diverse protocols employed in the limited studies reviewed, aiming to guide future research and inform shared decision-making in specific cases. For acute ankle sprains, the initial step involves determining the necessity of surgery vs conservative treatment. Engaging in shared decision-making with patients and respecting their treatment preferences is crucial. For patients opting for conservative treatment, initial management can include the RICE protocol. Drawing from the protocols used in the reviewed studies, PRP injections of 1.5–6 mL from 30–50 mL of blood extracts can be administered into the injured ligament under ultrasound guidance within the first 11 days after injury and again between 1 and 4 weeks post-injury. Unlike PRP, HA is administered periarticularly rather than intra-ligamentously. Consistent with Petrella et al.’s study, periarticular HA injections (0.7–1.2 mL; MW 750–1000 kDa; 20 mg) can be administered within 48 h of injury and repeated on day 4. However, these parameters are derived from individual studies with small sample sizes and should be considered exploratory rather than evidence-based recommendations. Notably, while evidence-based guidelines generally advocate for functional support over rigid immobilization for acute ankle sprains [1, 10], all studies evaluating regenerative therapies in our review employed immobilization protocols ranging from 3 days to 4 weeks [12–17]. This discrepancy may reflect concerns regarding protection of the injection site during the early healing phase or simply represent the protocols chosen by individual research teams. The potential for functional support to be safely and effectively combined with PRP or HA injections—offering the benefits of both regenerative therapy and early mobilization—remains a significant question for future research. The clinical algorithm presented in Figure 1 summarizes the diverse protocols used in the reviewed studies and should be interpreted as a hypothesis-generating framework to guide future research rather than a standardized treatment recommendation. Clinicians should tailor treatment decisions based on injury severity, patient activity levels, available evidence, institutional protocols, and thorough discussions with patients regarding the limited and preliminary nature of the evidence.

Multiple high-quality studies, including meta-analyses, systematic reviews, and randomized controlled trials, have assessed the efficacy and safety of PRP, HA, and their combination in the treatment of KOA [30–35]. It is important to recognize that the pathophysiology of chronic degenerative joint disease fundamentally differs from that of acute ligamentous injury, making direct extrapolation from KOA to acute ankle sprains inappropriate. However, we briefly discuss the KOA literature here to illustrate the biological plausibility and safety profile of combination regenerative therapy, rather than to support clinical recommendations for acute ankle sprains. Research on KOA indicates that PRP injections provide superior pain relief and functional improvement compared to placebo, corticosteroids, and HA alone, without increasing AEs [30]. Furthermore, the combination of PRP with HA has demonstrated greater clinical benefits than monotherapy, leading to enhanced pain reduction and improved joint function [31, 32]. These findings suggest a potential synergistic effect of combination therapy, although its applicability to acute ligamentous injury remains uncertain. Given the established benefits of combining PRP and HA in KOA treatment, we aimed to explore whether a similar approach could be effective for acute ankle sprains. However, a comprehensive search of available databases revealed no clinical trials specifically investigating the combined use of PRP and HA for this condition. This observation serves as a hypothesis-generating insight and highlights a promising area for future research. Importantly, the discussion of PRP and HA therapy in KOA is presented solely in an analogical context to illustrate the potential of combination regenerative therapy; it does not influence our treatment algorithms or conclusions regarding acute ankle sprains, which are based exclusively on six ankle-sprain-specific studies [12–17].

The five studies identified on PRP therapy for acute ankle sprains exhibited heterogeneous preparation protocols and clinical outcomes. PRP is not a standardized product; its composition varies significantly depending on preparation methods. Established classification systems, such as the Platelets, Activation, White cells (PAW) classification [36], the DEPA (Dose of injected platelets, Efficacy of the production method, Purity of the PRP, Activation process) classification [37], and the Mishra classification system [38], have been developed to characterize PRP preparations. These frameworks categorize PRP based on platelet concentration, leukocyte content, activation method, and preparation technique, all of which may influence biological activity and clinical outcomes. Among the studies included in this narrative review, some utilized leukocyte-rich PRP (LR-PRP) while others employed leukocyte-poor formulations, with activation methods ranging from autologous thrombin to calcium chloride or no activation. This heterogeneity in PRP composition represents a significant limitation in comparing results across studies and may partially account for the variable clinical outcomes observed. Beyond its role in managing acute ankle sprains, the application of PRP therapy in CAI remains controversial. The discussion of PRP therapy in CAI is outside the scope of the present study; it is included to explore other applications of regenerative therapy and will not inform our treatment algorithm or conclusions regarding acute ankle sprains. A retrospective study evaluated the safety and efficacy of PRP injections in patients with chronic lateral ankle instability [39]. PRP was injected into the injured talofibular ligaments over three sessions at 7-day intervals. Clinical and functional outcomes were assessed using the Karlsson score, Cumberland Ankle Instability Tool, Good’s grading system, patient satisfaction, and return-to-exercise time, revealing significant improvements in the Cumberland Ankle Instability Tool and Karlsson scores at 3 months (*P* < 0.000). The mean follow-up period was 17.94 ± 3.25 weeks, with no reported AEs, suggesting promising short-term benefits of PRP [39]. In contrast, a randomized controlled trial examined the effectiveness of LR-PRP injections in patients who underwent Modified Broström–Gould surgery for chronic lateral ankle instability. Forty patients were randomized into two groups: one group received standard postoperative management along with three ultrasound-guided LR-PRP injections, while the control group received only standard postoperative management. Although both groups exhibited significant improvements in the VAS and AOFAS scores at 6 months (*P* < 0.001), no significant differences were observed between the PRP and control groups regarding pain relief, function, or range of motion. The study concluded that LR-PRP did not provide additional clinical or functional benefits compared to conventional postoperative management [40]. While PRP and HA may offer benefits for CAI, addressing the underlying causes of CAI through surgical or other appropriate treatments remains essential [41]. These CAI data are presented solely for contextual purposes and are outside the scope of the present review; they are not used to inform our treatment algorithm or conclusions regarding acute ankle sprains.

This work presents several significant limitations that must be acknowledged. First, the evidence base is notably limited, comprising only five articles on PRP therapy and one on HA therapy that meet our inclusion criteria. This small number of studies precludes meta-analytic synthesis and constrains the strength of any conclusions drawn. Second, our search was confined to a single database (PubMed) and English-language publications, which may have introduced selection bias and excluded relevant studies published in other languages or indexed in alternative databases. While this approach ensured consistency in data extraction and interpretation of specialized clinical terminology, it represents a notable methodological limitation.

Third, all included studies featured small sample sizes (10–50 participants per group), which limits both statistical power and generalizability. Fourth, substantial heterogeneity exists across studies concerning patient populations (general vs athletic), injury severity, PRP preparation protocols, HA formulations, injection techniques, concurrent treatments (such as immobilization duration and NSAID use), and outcome measures. In particular, the reviewed studies on PRP employed varying preparations distinguished by platelet concentration, leukocyte content, and activation methods—parameters that can be characterized using established classification systems, including PAW, DEPA, and Mishra classifications [36–38]. This heterogeneity in PRP composition may significantly impact clinical outcomes, as different formulations exhibit distinct biological properties and inflammatory profiles, complicating direct comparisons and synthesis of results.

Fifth, most studies had short follow-up periods (typically 3–6 months), providing limited information on long-term efficacy, safety, or prevention of CAI. Sixth, only one study was placebo-controlled [16], and it reported no benefit of PRP over placebo, raising concerns about potential placebo effects in the uncontrolled studies. Finally, as a narrative review rather than a systematic review, our work lacks the methodological rigor associated with pre-registered protocols, risk-of-bias assessment tools, and meta-analytic synthesis. These limitations considerably diminish the certainty of the evidence and emphasize that our findings should be regarded as preliminary and hypothesis-generating rather than definitive.

Our findings align with and extend the only prior systematic review [12] specifically evaluating PRP for acute ankle sprains. That review concluded that PRP demonstrates potential benefits for short-term pain reduction and functional improvement but highlighted substantial heterogeneity in PRP preparation protocols, limited long-term data, and the necessity for high-quality randomized controlled trials with standardized protocols. Our narrative review corroborates these conclusions and additionally underscores the following: (1) the single placebo-controlled trial [16] found no benefit of PRP, suggesting potential publication bias or placebo effects in uncontrolled studies; (2) the extremely limited evidence for HA (only one randomized controlled trial (RCT) [17]), which has received less attention than PRP; (3) the complete absence of studies examining combination PRP and HA therapy for acute ankle sprains, despite promising results in other conditions; and (4) significant methodological inconsistencies across studies, including variable immobilization protocols that conflict with evidence-based guidelines recommending functional support.

While our review offers a broader synthesis encompassing both PRP and HA and provides a hypothesis-generating treatment framework, we emphasize the same cautionary conclusions as the prior systematic review: current evidence remains insufficient to support the routine clinical use of regenerative therapies for acute ankle sprains outside of research settings or carefully selected cases involving thorough patient counseling. Despite these limitations, we conclude that PRP and HA may serve as adjuvant therapies for acute ankle sprains in selected cases; however, the evidence base remains limited and preliminary. Further high-quality randomized controlled trials with standardized PRP preparation, injection protocols, and long-term follow-up are essential to establish definitive recommendations.

## Conclusion

The low-certainty evidence from a limited number of small studies suggests that PRP or HA may provide short-term symptomatic relief as adjuvant therapies for acute ankle sprains, particularly within athletic populations. However, substantial limitations exist, including small sample sizes, heterogeneous protocols, inconsistent outcome measures, and short follow-up periods, with the only placebo-controlled trial indicating no benefit of PRP over placebo. These limitations preclude definitive conclusions regarding efficacy or optimal protocols. Robust, standardized, multicenter randomized controlled trials with adequate sample sizes, standardized preparation protocols, validated outcome measures, long-term follow-up (minimum 12–24 months), and appropriate controls are necessary before routine clinical adoption. Future research should investigate combination therapies, optimal dosing and timing, cost-effectiveness, and long-term prevention of CAI. Until high-quality evidence is available, regenerative therapies should be considered investigational and limited to research settings or carefully selected cases with thorough patient counseling.

## Data Availability

No datasets were generated or analyzed during the current study.
